# On the Distribution of Tract Lengths During Adaptive Introgression

**DOI:** 10.1534/g3.120.401616

**Published:** 2020-08-05

**Authors:** Vladimir Shchur, Jesper Svedberg, Paloma Medina, Russell Corbett-Detig, Rasmus Nielsen

**Affiliations:** * Department of Integrative Biology and Department of Statistics, UC Berkeley, Berkeley, CA; † National Research University Higher School of Economics, Russian Federation; ‡ Department of Biomolecular Engineering and Genomics Institute, UC Santa Cruz, California 95064; § Center for GeoGenetics, Globe Institute, University of Copenhagen, 2100 Denmark

**Keywords:** adaptive introgression, tract length, adaptation, *EPAS1*, admixture, selection

## Abstract

Admixture is increasingly being recognized as an important factor in evolutionary genetics. The distribution of genomic admixture tracts, and the resulting effects on admixture linkage disequilibrium, can be used to date the timing of admixture between species or populations. However, the theory used for such prediction assumes selective neutrality despite the fact that many famous examples of admixture involve natural selection acting for or against admixture. In this paper, we investigate the effects of positive selection on the distribution of tract lengths. We develop a theoretical framework that relies on approximating the trajectory of the selected allele using a logistic function. By numerically calculating the expected allele trajectory, we also show that the approach can be extended to cases where the logistic approximation is poor due to the effects of genetic drift. Using simulations, we show that the model is highly accurate under most scenarios. We use the model to show that positive selection on average will tend to increase the admixture tract length. However, perhaps counter-intuitively, conditional on the allele frequency at the time of sampling, positive selection will actually produce shorter expected tract lengths. We discuss the consequences of our results in interpreting the timing of the introgression of *EPAS1* from Denisovans into the ancestors of Tibetans.

Admixture—a process wherein genetically distinct populations hybridize and exchange alleles—is increasingly recognized as a dominant force in evolutionary genomics. In particular, admixture has the potential to introduce adaptive alleles from a donor population to a recipient, particularly if the donor population has already adapted to *e.g.*, unique environmental conditions. For example, when the ancestors of modern ethnic Tibetans first colonized the extremely high elevation Tibetan Plateau their success in adapting to these harsh conditions stemmed in part from genes acquired from a Denisovan-like ancestral population (Huerta-Sánchez *et al.* 2014). More generally, as genome sequence data continues to accumulate, it is increasingly clear that introgression has played a major role in shaping adaptive outcomes across a wide range of species, *e.g.*, (Hufford *et al.* 2013; Martin *et al.* 2013). However, the specific genetic signature of adaptive introgression and its impact on patterns of genetic variation remains less explored (but see *e.g.*, (Setter *et al.* 2019)). There is, therefore, a clear need to develop a theoretical framework to investigate the impacts of adaptive introgression on the genomes of natural populations.

When two ancestral populations hybridize, the genome of each individuals is a mixture of the ancestral populations, and the ancestry at each site in the genome can be traced back to a single population. Because alleles from a given ancestral population are initially on the same chromosomes, and therefore strongly linked within the admixed population, this process can dramatically shape linkage disequilibrium within admixed populations (commonly termed admixture LD (Stephens *et al.* 1994; Falush *et al.* 2003)). Admixture LD, and the haplotypic analog, ancestry tracts, which are unbroken stretches of sites from one ancestral population, have become the key elements of genomic inference in studying admixed populations. These two features of genetic variation are the primary units of demographic model inference within admixed populations. For example, the decay of LD (Loh *et al.* 2013) and the ancestry tract length distribution (Gravel 2012; Corbett-Detig and Nielsen 2017) (Skov *et al.* 2018) are commonly used for timing the onset of admixture assuming a neutral model during admixture.

(Hill 1974; Liang and Nielsen, 2014b 2016) showed recombination, genetic drift, and migration processes act as linear operators on the 2-locus and 3-locus linkage disequilibrium coefficients (as defined in (Lewontin and ichi Kojima 1960) and (Slatkin 1972) respectively). In contrast, natural selection is not a linear operator on the LD coefficients, and these approaches can, therefore, not be extended to accommodate the impacts of natural selection. Instead, other methods are required to analyze the impact of selection. A recent study (Sachdeva and Barton 2018) derives the ancestry tract length distribution under an infinitesimal selection model. Such a model assumes that there are many different sites and each of them is under weak selection. However, another potent force that affects admixed populations is adaptive introgression, where one or a few adaptive genes is acquired via hybridization and each has potentially strong effects on fitness. The analysis of adaptive introgression and its impacts on linkage disequilibrium and the ancestry tract length distribution is a largely unexplored topic and requires a novel theoretical framework.

The distribution of tract lengths during adaptive introgression is closely related to the study of the strength of linkage disequilibrium. Two main models that address this question are 2-locus and 3-locus linkage models, which model the joint behavior of 2 and 3 linked loci respectively. Substantial theory and numerical simulations are known from earlier works, *e.g.*, (Kimura 1956; Maynard and Haigh 2007; Thomson 1977; Lewontin and ichi Kojima 1960; Ewens 1968; Karlin and Feldman 1970; Feldman *et al.* 1974; Karlin and Carmelli 1975; Karlin 1975; Lewontin, 1964a, b; Franklin and Lewontin 1970)). There are two main differences between these works and our approach. The first is the problem itself: in the aforementioned works the object of interest is primarily the strength of LD in different selection scenarios, so all the sites are considered to be segregating in a single ancestral population, and allele frequencies at all the sites affect the strength of LD. In this paper we are instead interested in the length of an admixture tract covering a selected site. The second difference is that aforementioned works consider forward time models, and we consider coalescent models.

There are many other works which have used coalescent theory to study the dynamics of selective sweeps. In particular, (Kaplan *et al.* 1989) examine how the number of polymorphic sites changes due to selective sweeps using a deterministic logistic approximation to the selected allele frequency trajectory. (Barton 1998) expanded on this by considering the stochastic phase of the allele frequency trajectory when the allele frequency is close to 0 or 1. He showed that conditioning on the selected allele not being lost, its spread in the population is faster than exponential. (Durrett and Schweinsberg 2004) also showed, through simulations, that the logistic approximation performs poorly if the allele frequency is close to the boundaries (0 or 1).

The distribution of ancestry tract lengths around a locus under selection can reveal the strength of selection and the timing of admixture without requiring the assumption of neutrality. In this work, we present an efficient way to calculate the expected ancestry tract length distribution, and we analyze the dependence of this distribution on a range of different biological parameters. In particular, it is evident that for a given admixture proportion, selection results in longer tract lengths of introgressed segments. On the other hand, when conditioning on the allele frequency at the time of sampling, positive selection actually results in shorter ancestry tracts during adaptive introgression.

## Theory and Methods

### Conceptual overview

We model the tract length distribution around a selected locus. In other words, we want to find the probability that an ancestry tract ends at a certain distance from the selected locus because of a recombination event within the admixed population. We imagine a stochastic process along the length of the chromosome where transitions between ancestries at a given locus are caused by recombination (at this locus) such that chromosomes on the left and on the right of the recombination site are derived from different ancestral populations. As shown in (Thomson 1977), the strength of linkage disequilibrium between two neutral loci depends on the distance of the region containing those loci from the selected site. Similarly, transition rates between ancestry types are not uniform in recombination units with regards to the distance from the selected site.

Indeed, if a neutral locus is far away from the selected locus, the surrounding genomic region is nearly independent of the impact of selection, and the transitions between the ancestry types should occur almost as expected under neutrality. On the other hand, if a given site is tightly linked to a selected locus, the probability of transitions to a different ancestry would be affected by the allele frequency changes in the selected locus. Because a 2-locus framework cannot model this dependency, it does not provide enough information regarding the local transition rates in genomic regions proximal to a selected site. Instead, in this work, we use a 3-locus model to calculate transition rates between different ancestries in two loci as a function of the distance to a third selected locus.

### Selected allele trajectory

Let *α* denote a locus under selection with two possible alleles *A* standing for the selected allele and *a* standing for neutral allele. We are interested in the scenario when allele *A* is introduced into the population through an adaptive introgression event that includes instantaneous replacement of a given proportion of individuals, the admixture fraction, in the recipient population. Such an introgression event is commonly termed an “ancestry pulse” in related works, *e.g.*, (Gravel 2012; Corbett-Detig and Nielsen 2017). We assume that allele *A* was fixed in the donor population, and that prior to admixture, allele *a* was fixed in the recipient population.

The expected trajectory of an allele under selection can be well approximated using a logistic function (see *e.g.*, (Kaplan *et al.* 1989)) under the following conditions: First, selection should be strong ($N_{e}s\gg 1$, where $N_{e}$ is effective population size, and *s* is selection coefficient such that an individual with two selected alleles has fitness 1+s and a heterozygous individual has fitness 1+s/2). Second, the frequency of the selected allele is above a critical threshold, so in our model, the admixture fraction must not be too small. Finally, the time since adaptive introgression is not too large compared to the selection coefficient, so that the frequency of *A* is not too close to 1 at the time of sampling (Smith 1971; Messer and Neher 2012).

Approximating the allele frequency trajectory of the adaptive allele using a deterministic logistic trajectory, as in (Kaplan *et al.* 1989), allows us to avoid integration over the stochasticity caused by genetic drift, which makes our approach similar to the mean field theory widely used in physics (Weiss 1907) and epidemiology (Kermack William Ogilvy and Thomas 1927). We show that this approximation is highly accurate, in a certain range of parameters, for estimating the expected tract lengths. In this range, the expected tract length can be considered invariant under the order of integration over genetic drift and over the tract length distribution.

Outside this range, when the proportion of introgression is very small, which means that drift is strong relative to selection, the logistic function will yield inaccurate predictions. This observation matches that of (Durrett and Schweinsberg 2004). In this case the expected trajectory can be estimated by generating a large random sample of stochastic trajectories. This can be done efficiently, because, in practice, the selection coefficient s≪1 is small, and the diploid population can be approximated by a haploid population under genic selection. At each generation the selected allele frequency is simulated from a binomial distribution with probability weighted by the total fitness contributed of the selected allele. We average allele frequencies at each generation over the simulated trajectories (conditioning on observing the selected allele at the time of sampling). We will show that such an approach vastly extends the applicability of our method to cases that require greater stochasticity. In particular, we use it to analyze the Denisovan introgression in the ancestry of Tibetans, where the proportion of introgression is estimated to be as small as 0.06% (Huerta-Sánchez *et al.* 2014), and therefore is outside the range of parameters where the logistic approximation to the allele frequency trajectory yields accurate results.

### 3-locus model during adaptive introgression

Let *β* and *γ* be two neutral loci near *α* (the locus under selection) so that there are 3 loci on the chromosome: α,β, and *γ*, following each other sequentially in this order on the chromosome. Let $r_{1}$ be the distance (in Morgans) between *α* and *β*, $r_{2}$ is the distance between *β* and *γ*. We are following the ancestry of *β* and *γ*. In other words, we track the lineage leading to each locus to the time of introgression, while simultaneously tracking how recombination and coalescence act on the segment. If at the time of introgression the ancestral locus is on a haplotype carrying allele *A*, then, by definition, its ancestry is from the introgressed population (denoted ancestry type 1). If, in contrast, the ancestral locus is linked to allele *a*, then it comes from the recipient population (denoted ancestry type 0). Ancestry type 0 corresponds to the recipient population and ancestry type 1 corresponds to the donor population.

The importance of this 3-locus model is that we can calculate transition rates between ancestry types, and hence, we can numerically calculate the distribution of tract lengths of ancestry type 1 (or type 0) near the locus *α*.

### Model derivation

Under the coalescent with recombination, the model can be considered Markovian backward in time, when conditioned on the allele frequency path. To describe the dynamics of the adaptive introgression 3-locus model, we need to enumerate the possible states, which describes the ancestry configurations across the three loci, and we need to find the transition rates between them.

The model has 6 possible states. Each state represents an ancestral configuration for an observed chromosome with three loci: *α*, *β* and *γ*. At *α* we track the allelic state, *A* or *a*, which also indicates ancestry. In *β* and *γ* we only need to know if the given chromosome is ancestral to the observed chromosome or not. We use the notation $\beta^{a}$ and $\gamma^{a}$ to indicate DNA in *β* or *γ* that is ancestral to the observed chromosome. $\beta^{n}$ and $\gamma^{n}$ is used to indicate DNA that is not ancestral to the observed chromosome. The six states are then:

- $\left(A-\beta^{a}-\gamma^{a}\right)$: ancestral material for the observed chromosome at *β* and *γ* on a chromosome carrying allele *A* at the selected locus *α*,
- $\left(A-\beta^{a}-\gamma^{n},A-\beta^{n}-\gamma^{a}\right)$: ancestral material at *β* and *γ* on different chromosomes, both carrying allele *A*,
- $\left(A-\beta^{a}-\gamma^{n},a-\beta^{n}-\gamma^{a}\right)$: ancestral material at *β* and *γ* on different chromosomes, and the chromosome ancestral to the observed chromosome at *β* is carrying allele *A*, while the chromosome ancestral to the observed chromosome at *γ* is carrying allele *a*,
- $\left(a-\beta^{a}-\gamma^{n},A-\beta^{n}-\gamma^{a}\right)$: ancestral material at *β* and *γ* on different chromosomes, and the ancestral chromosome at *β* is carrying allele *a*, while the ancestral chromosome at *γ* is carrying allele *A*,
- $\left(a-\beta^{a}-\gamma^{n},a-\beta^{n}-\gamma^{a}\right)$: the ancestral material of *β* and *γ* are on different chromosomes, both carrying allele *a*,
- $\left(a-\beta^{a}-\gamma^{a}\right)$: ancestral material at both *β* and *γ* are on the same chromosome carrying allele *a*.

We denote the frequency of the selected allele at time *t* by ω(t). As we indicated previously, we assume that ω(t) deterministically follows a logistic function:

$$\omega \left(t\right)=1-\frac{1}{1+e^{-st/2}}=\frac{1}{1+e^{st/2}},$$

because we are working in backward time. This is a reflection of a logistic function relative to t=0. The time of introgression corresponds to a point, $t_{1}$, on the deterministic allele frequency trajectory such that $\omega \left(t_{1}\right)$ equals the admixture proportion $\omega_{1}$. Notice, that the time of sampling $t_{0}=t_{1}-T$, where *T* is the time since introgression, does not necessarily equal t=0.

Recombination acts at a rate proportional to the recombination distances between loci. We assume that recombination between loci *α* and *β* occurs at rate $r_{1}$ and recombination occurs between loci *β* and *γ* at rate $r_{2}$. In other words, we measure distance between the loci in Morgans.

Consider for example the ancestry configuration state $\left(A-\beta^{a}-\gamma^{a}\right)$ at time *t*. If recombination occurs between loci *α* and *β*, then ancestral material at loci *β* and *γ* remain on the same ancestral chromosome. The allele at the site *α* on this ancestral chromosome will be *A* with probability ω(t) (state does not change) and *a* with the probability 1−ω(t). Hence, the transition rate from state $\left(A-\beta^{a}-\gamma^{a}\right)$ to the state $\left(a-\beta^{a}-\gamma^{a}\right)$ is $r_{1}\left(1-\omega \left(t\right)\right)$ at time *t*. If a recombination event occurs on a chromosome with configuration $\left(A-\beta^{a}-\gamma^{a}\right)$ between loci *β* and *γ*, then it is split into two chromosomes, and there are two different possible ancestry configurations. In both configurations the first ancestral chromosome is $A-\beta^{a}-\gamma^{n}$. The second chromosome is either $A-\beta^{n}-\gamma^{a}$ with probability ω(t), or $a-\beta^{n}-\gamma^{a}$ with probability 1−ω(t). So, recombinations are the first type of transition in our model. The other type of transition is coalescence, which is possible between chromosomes that carry the same allele (*A* or *a*) at the selected locus *α* at the rate λ/ω(t) for *A* and $\lambda /\bar{\omega }\left(t\right)$ for *a*, where $\lambda =1/2N_{e}$ and $2N_{e}$ is the haploid effective population size. The full transition rate matrix is

$$M(t)=\left(\begin{array}{cccccc} -r_{1}\bar{\omega }(t)-r_{2} & r_{2}\omega (t) & r_{2}\bar{\omega }(t) & 0 & 0 & r_{1}\bar{\omega }(t)\\ \lambda /\omega (t) & -\lambda /\omega (t)-(2r_{1}+r_{2})\bar{\omega }(t) & \left(r_{1}+r_{2})\bar{\omega }(t\right) & r_{1}\bar{\omega }(t) & 0 & 0\\ 0 & \left(r_{1}+r_{2})\omega (t\right) & -r_{1}-r_{2}\omega (t) & 0 & r_{1}\bar{\omega }(t) & 0\\ 0 & r_{1}\omega (t) & 0 & -r_{1}-r_{2}\bar{\omega }(t) & \left(r_{1}+r_{2})\bar{\omega }(t\right) & 0\\ 0 & 0 & r_{1}\omega (t) & \left(r_{1}+r_{2})\omega (t\right) & -\lambda /\bar{\omega }(t)-(2r_{1}+r_{2})\omega (t) & \lambda /\bar{\omega }(t)\\ r_{1}\omega (t) & 0 & 0 & r_{2}\omega (t) & r_{2}\bar{\omega }(t) & -r_{1}\omega (t)-r_{2} \end{array}\right),$$

where $\bar{\omega }\left(t\right)=1-\omega \left(t\right)$ is the allele frequency of allele *a*, and the equation describing the probability of being at a certain state at time *t* is

ℙ′(t)=ℙ(t)M(t). (1)

The initial condition for this differential equation is

$$\mathbb{P}\left(t_{0}\right)=\left(1,0,0,0,0,0\right)$$

for the dynamics of introgressed tracts (hence, carrying allele *A*), and

$$\mathbb{P}\left(t_{0}\right)=\left(0,0,0,0,0,1\right)$$

for the tracts from the recipient population (individuals with allele *a*).

Equation 1 cannot be solved analytically, because eigenvalues of the matrix M(t) cannot be derived analytically in general. However, it is easy to solve it with standard numerical methods. Our implementation uses scipy.integrate.odeint() (Jones *et al*. 2001) which is based on lsoda (Hindmarsh 1983) from the FORTRAN library odepack.

Again, we assume that allele *a* is private to the recipient ancestral population, and allele *A* is private to the donor ancestral population. To estimate the ancestry of loci *β* and *γ*, it is necessary to trace the ancestral chromosomes to the time of introgression $t_{1}$. If ancestral locus appears on a chromosome with allele *a* (allele *A*) at that time we conclude that it comes from the recipient population (donor population respectively), hence it has ancestry type 0 (ancestry type 1 respectively). Our goal is to calculate the expected length of the introgression tract containing the locus under selection, *i.e.*, haplotypes with allele *A*. The fact that the tract ends at a certain position on a genome means that all loci (*β*) to the left of this position has ancestry type 1 and a loci (*γ*) to the right of this position has ancestry type 0. Moreover, all the loci between *α* and *β* have ancestry type 1, and *β* and *γ* are infinitesimally close to each other. The mathematical derivation of this idea is in the next subsection. We also note that this process is a type of of Sequentially Markov Coalescent (SMC) model (McVean and Cardin 2005) which considers two Markovian process: backward-in-time coalescent process and along-the-genome Markov chain which generates recombination breakpoints.

### Transition rates between ancestry of type 0 and type 1

As previously discussed, we are interested in transitions of ancestry in loci near the introgressed selected allele. More precisely, we seek the distribution of lengths of ancestry tracts that contain *A*, *i.e.*, the set of ancestry tracts with ancestry type 1 at the selected site. In the given model the probability that a locus is of ancestry type 1 or type 0 is equal to the probability that the ancestral chromosome carries the allele *A* or *a* respectively at time of introgression $t_{1}$. In the previous subsection we described a Markovian process (with regards to the backward time) under which we can calculate those probabilities.

Now we consider a new Markov process, which describes the ancestry at a locus while moving along the chromosome away from the selected locus. The process is only approximate because the real process along the length of the chromosome is not Markovian. The states of this Markov process are again ancestries of type 0 or 1. By definition, transition rate between states $s_{1}$ and $s_{2}$ at position *r* of a Markov process S(r) is

$$\tau_{s_{1},s_{2}}\left(r\right)=\underset{dr\to 0}{\text{lim}}\frac{P(S\left(r+dr\right)=s_{2}|S\left(r\right)=s_{1})}{dr}.$$

The transition rate is $\tau_{10}\left(r\right)$ between ancestries of type 1 and type 0, which corresponds to recombination breaking the introgressed ancestry tract, is

$$\tau_{10}\left(r\right)=\underset{dr\to 0}{\text{lim}}\frac{P(S\left(r+dr\right)=0|S\left(r\right)=1)}{dr}=\underset{dr\to 0}{\text{lim}}\frac{1}{dr}\frac{P\left(S\left(r+dr\right)=0,S\left(r\right)=1\right)}{P\left(S\left(r\right)=1\right)}.$$ (2)

The numerator P(S(r+dr)=0,S(r)=1) is the probability of being at the third state $\left(A-\beta^{a}-\gamma^{n},a-\beta^{n}-\gamma^{a}\right)$, and the denominator P(S(r)=1) is the marginal probability of ancestry type 1, hence it is the sum of probabilities of the first three ancestral configurations $\left(A-\beta^{a}-\gamma^{a}\right)$, $\left(A-\beta^{a}-\gamma^{n},A-\beta^{n}-\gamma^{a}\right)$ and $\left(A-\beta^{a}-\gamma^{n},a-\beta^{n}-\gamma^{a}\right)$. Expression 2 is easy to evaluate numerically by considering sufficiently small values of $r_{2}$.

### Model validation

In order to estimate how accurate our deterministic approximation is, we compared our results with the average tract length estimated from simulations using the forward-in-time simulation framework SELAM (Corbett-Detig and Jones 2016). Briefly, we used the software to simulate a Wright-Fisher population of constant size 5000 hermaphroditic diploid individuals. We simulated a single chromosome of length one Morgan with a single selected site at position 0.5 Morgans. At each sampling point, we extracted 50 individuals (100 chromosomes) at random from within the population and output the ancestry across each chromosome. We performed 10 to 100 thousand replicates of each combination of selective coefficient, times since admixture and admixture proportion.

### Data availability

The authors state that all data necessary for confirming the conclusions presented in the article are represented fully within the article. The code is available at the GitHub repository associated with this project https://github.com/vlshchur/DAIM.

## Results

### Accuracy of deterministic approximation

We simulate both relatively weak and strong selection, and we allow selection to act in different periods of time. We mostly explore the accuracy through the comparison of expected tract length and its variance. We also present the distribution of break-points of tracts containing selected allele. The tract length is the sum of two independent random variables, one representing the distance to the left of the selected mutation and the other representing the distance to the right of the selected mutation.

**Figure 1:**
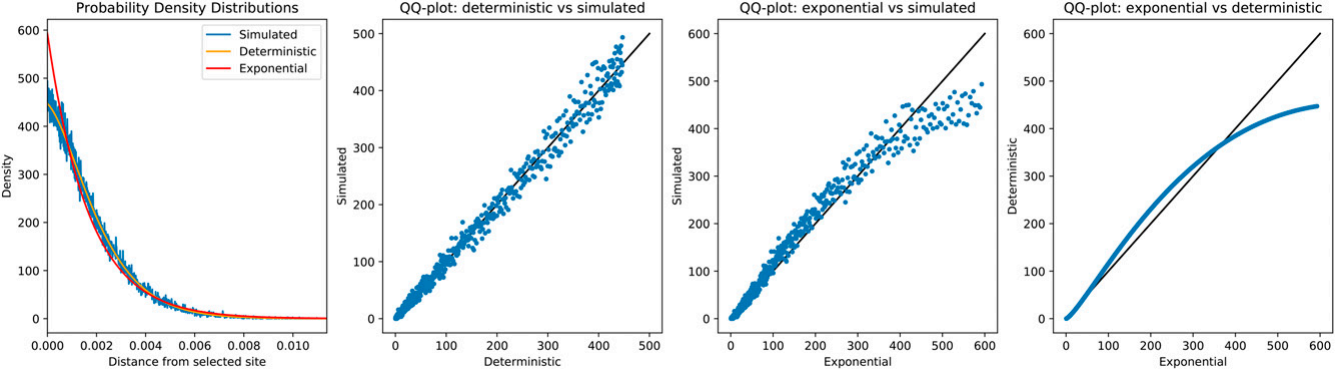
Distribution of the distance from the selected locus to one end of the introgressed tract. Selection coefficient s=0.01, admixture fraction $\omega_{1}=0.1$ and time since introgression T=1000 generations. The observed allele frequency is $\omega_{0}=0.94$. The first panel shows the probability density functions for the empirical distribution obtained by simulations, the distribution calculated under the deterministic approximation and the exponential distribution with the mean set equal to the simulated mean. Three other panels are qq-plots showing all three pairs of the presented distributions.

As the waiting time to a recombination event along the length of the genome is exponentially distributed, one might be inclined to think that tracts lengths also are exponentially distributed. However, this is not the case as illustrated in Figure 1. The figure shows the distribution of the distance from the selected locus to one end of the tract, estimated from simulations, from the deterministic approximation, and an exponential distribution with the rate equal to the inverse of the mean of the simulated distribution. In this scenario, the approximation provides an accurate estimate of the empirical distribution. The exponential distribution provides a much worse fit. The reason why the exponential distribution fits so poorly is that it does not incorporate the possibility of back-recombination and coalescence. In the presence of these processes, the tract length distribution is not exponential (see also (Liang and Nielsen, 2014a) for more discussion of this).

In all of the scenarios that we explored, the relative error does not exceed 5%, and in more than half of all cases it is within 2% (Tables 1 and 2). This level of precision should be sufficient for most applications given that the uncertainty in the real data analysis is usually much greater than the error of the approximation model.

**Table 1:** **The accuracy of the deterministic approximation for the expected tract length under adaptive introgression compared to estimates from simulations. For every set of parameters (introgression fraction, selection coefficient, and time of introgression), we performed** $10^{5}$ **replicate= simulations. The haploid effective population size was 10,000 chromosomes, with 100 chromosomes sampled from each population. The relative error was calculated by comparing the simulated expected tract length to the prediction given by the deterministic model.**

Introgression parameters			Expected tract length		
Proportion	Selection	Time	Simulations	Deterministic approximation	Relative error
0.01	0.001	50	0.0417436	0.0401907	3.7%
		100	0.0197123	0.0200985	2.0%
		500	0.00407317	0.00402525	1.1%
		1000	0.00206252	0.00201644	2.2%
0.01	0.01	50	0.0415686	0.0402287	3.3%
		100	0.0202129	0.02014	0.4%
		500	0.00423004	0.00411598	2.8%
		1000	0.00237283	00227902	4.0%
0.05	0.001	50	0.0427474	0.0419025	2.0%
		100	0.0210069	0.0209646	0.2%
		500	0.00428813	0.00421551	1.7%
		1000	0.00217073	0.00212356	2.2%
0.05	0.01	50	0.0428456	0.0420981	1.7%
		100	0.021438	0.0211766	1.2%
		500	0.00470731	0.00462923	1.7%
		1000	0.00290033	0.00286404	1.3%

**Table 2:** **The accuracy of the deterministic approximation for the expected tract length under adaptive introgression compared to the estimates from simulations for numerically estimated trajectory. For scenarios with relatively small admixture fractions (**$\omega_{1}$ **= Proportion = 0.0006), the logistic function does not accurately describe the allele frequency trajectory, so we numerically estimated the mean trajectory using stochastic simulations. The relative error is for the deterministic approximation with numerically estimated trajectories relatively to the simulation estimates.**

Introgression parameters			Expected tract length			Relative error
				Deterministic	Deterministic	
Proportion	Selection	Time	Simulations	Approximation	Approximation	
				(numerical)	(logistic)	
0.0006	0.01	1500	0.00176	0.00173	0.00142	1.7%
		1750	0.00158	0.00160	0.00129	1.3%
		2000	0.001503	0.001500	0.00120	0.2%
		2250	0.00141	0.00142	0.00114	0.7%
0.0006	0.02	1500	0.00234	0.00230	0.00198	1.7%
		1750	0.00222	0.00215	0.00186	3.2%
		2000	0.00210	0.00203	0.00175	3.3%
		2250	0.00205	0.00193	0.00167	5.9%
0.025	0.001	1500	0.001415	0.001436	0.00138	1.5%
		1750	0.001227	0.001244	0.001189	1.4%
		2000	0.001136	0.001101	0.001043	3.1%
		2250	0.001020	0.000990	0.000930	2.9%
		2500	0.000883	0.000902	0.000839	2.2%
0.025	0.005	1500	0.00164	0.00158	0.00155	4.9%
		1750	0.00146	0.00142	0.00139	2.7%
		2000	0.00133	0.00130	0.00128	2.3%
		2250	0.00126	0.00121	0.00120	4.0%
		2500	0.00118	0.00114	0.00114	3.4%

As previously discussed, the logistic function is a good approximation for the allele frequency trajectory only when selection is strong. Still, we wondered if the deterministic approach would give consistent results for a case of neutral admixture without adaptive introgression. Following Liang, Nielsen (Liang and Nielsen, 2014a) the expected tract length under a neutral admixture model can be calculated as

$$\frac{2}{2N_{e}\left(1-\omega_{1}\right)\left(1-e^{-T/2N_{e}}\right)}$$ (3)

under the SMC’ model (Marjoram and Wall 2006). Notice that there is a factor of two in the numerator due to the fact that we condition on observing the introgressed allele on a haplotype. We find that in the limiting case of no selection, the approximation closely approximates the analytical result from (Liang and Nielsen, 2014a) (Table 3).

**Table 3:** Deterministic prediction for the expected tract length in a neutral model compared to the theoretical expectation under SMC’ model.

Proportion	Time	Expected tract length (deterministic approximation)	Expected tract length (theoretical)	Relative error
0.01	10	0.20194	0.200923	0.5%
	100	0.0201956	0.0200946	0.5%
	1000	0.00202122	0.00201177	0.5%
0.05	10	0.210491	0.209387	0.5%
	100	0.021054	0.0209442	0.5%
	1000	0.00211024	0.0020999	0.5%
0.1	10	0.222252	0.222278	0.01%
	100	0.022233	0.0222778	0.2%
	1000	0.00223105	0.00227824	2.1%

Another case where the logistic function is not expected to be a good approximation of the mean trajectory, is when the admixture proportion is close to zero. To explore this issue, we chose a set of parameters, which are similar to Neanderthal introgression into non-Africans (2.5%), and to the Denisovan introgression into Tibetans (0.06%). We found that the logistic approximation performs poorly for the Denisovan-like scenario, and is not very accurate (up to 10% relative error) for the Neanderthal-like introgression with week selection (s=0.001); in particular, the predicted allele frequency is strongly underestimated. Presumably these discrepancies reflect the increased impacts of genetic drift for small admixture proportions. To address this issue, we estimated the expected trajectory by forward-in-time stochastic simulations of a large sample of possible allele frequency trajectories (see subsection Selected allele trajectory for details). The stochastically estimated expected trajectory is then used instead of the logistic function in Equation 1 to model the tract length distribution. The deterministic model (with numerically pre-computed expected trajectories) gives tract length estimates quite similar to those observed in the simulated data sets (Table 2).

### Variance

The variance in the tract length distribution predicted by our method is an accurate approximation of the across population variance, that is, the variance among all the tracts obtained in many replicates with the same parameters (see Table 4). This is equivalent to the variance in tract length among tracts within a population if the tracts introgressed into the population independently of each other and have not coalesced or recombined with each other. Of course, in reality, two tracts might coalesce before the time of introgression, so their lengths are not independent of each other. Hence, we expect that the within-population variance is smaller than across-population variance. On the other hand, if introgression is recent enough, then the chance of coalescence is small, and the correlation between tract lengths within population will be small.

**Table 4:** Simulated across and within population variance and the across population variance estimated from the deterministic model. The percents show the relative error of the deterministic approximation compared to the simulations both for across and within population values. The introgression with 0.0006 (or, 0.06%) admixture proportion was chosen to approximate Denisovan introgression into Tibetans. The introgression with 0.025 (or, 2.5%) admixture proportion was chosen to approximate Neanderthal introgression into non-Africans.

Introgression parameters			Standard deviation of tract length				
Proportion	Selection	Time	Deterministic approximation	Simulated (across populations)		Simulated (within population all replicates)	
0.01	0.001	50	0.028513	0.029397	3.01%	0.027405	4.04%
		100	0.014260	0.013484	5.75%	0.013376	6.61%
		500	0.002858	0.002795	2.25%	0.002334	22.45%
		1000	0.001433	0.001454	1.44%	0.001131	26.7%
0.01	0.01	50	0.028534	0.028719	0.64%	0.027571	3.49%
		100	0.014283	0.013762	3.79%	0.013481	5.95%
		500	0.002904	0.003050	4.79%	0.002621	10.8%
		1000	0.001548	0.001548	0.0%	0.001438	7.65%
0.05	0.001	50	0.029728	0.030492	2.51%	0.029590	0.47%
		100	0.014875	0.014932	0.38%	0.014642	1.59%
		500	0.002993	0.003035	1.38%	0.002757	8.56%
		1000	0.001509	0.001554	2.9%	0.001336	12.95%
0.05	0.01	50	0.029834	0.030620	2.57%	0.029731	0.35%
		100	0.014989	0.014980	0.06%	0.014830	1.07%
		500	0.003196	0.003246	1.54%	0.003120	2.44%
		1000	0.001835	0.001888	2.81%	0.001785	2.8%
0.0006	0.01	1500	0.001125	0.001173	4.09%	0.000962	16.94%
		1750	0.001014	0.000998	1.6%	0.000889	14.06%
		2000	0.000930	0.000954	2.52%	0.000811	14.67%
		2250	0.000864	0.000884	2.26%	0.000751	15.05%
0.0006	0.02	1500	0.001350	0.001423	5.13%	0.001241	8.78%
		1750	0.001230	0.001312	6.25%	0.001132	8.66%
		2000	0.001136	0.001221	6.96%	0.001050	8.19%
		2250	0.001061	0.001179	10.01%	0.000974	8.93%
0.025	0.001	1500	0.001015	0.001052	3.52%	0.000806	25.93%
		1750	0.000878	0.000840	4.52%	0.000677	29.69%
		2000	0.000776	0.000835	7.07%	0.000589	31.75%
		2250	0.000696	0.000691	0.72%	0.000535	30.09%
		2500	0.000633	0.000608	4.11%	0.000477	32.7%
0.025	0.005	1500	0.001082	0.001136	4.75%	0.000991	9.18%
		1750	0.000956	0.000970	1.44%	0.000875	9.26%
		2000	0.000863	0.000887	2.71%	0.000784	10.08%
		2250	0.000793	0.000836	5.14%	0.000726	9.23%
		2500	0.000737	0.000779	5.39%	0.000672	9.67%

Indeed, for most of the scenarios with 50 and 100 generations since the time of introgression (Table 4) the variances of the tract lengths are similar. For larger times, the across-populations and within-population variance are different by up to 20%, reflecting the increased probability of coalescence among selected haplotypes that share a recombination event within the admixed population.

### Run time

Calculation of the expected tract length for 100 introgression scenarios takes just 106 sec for a Python implementation executed on MacBook Pro (2.9 GHz Intel Core i5) when using the logistic function to approximate the allele frequency trajectory. Transition rates are evaluated at 1000 points (different values of *r*). The code is available at the GitHub repository associated with this project https://github.com/vlshchur/DAIM.

### Dependence of mean tract length on different parameters

**Figure 2:**
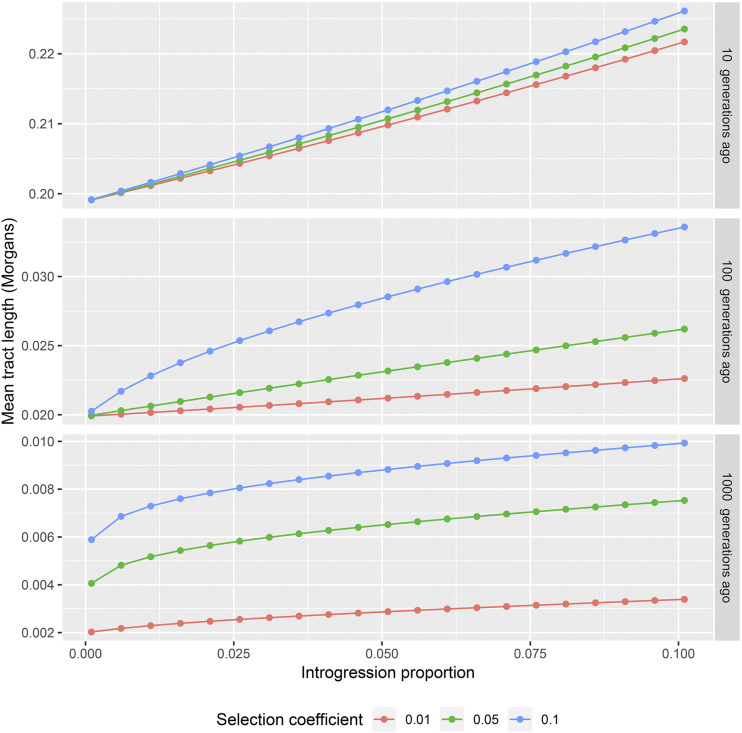
Dependence of the expected tract length on the proportion of introgression. Different panels correspond to different times of introgression (10, 100 and 1000 generations respectively). Different colors correspond to different selection coefficient values (0.01, 0.05, 0.1) Notice that as the time since introgression changes by an order of magnitude, the tract lengths change by an order of magnitude also. So, the y-axes here are shown on different scales.

**Figure 3:**
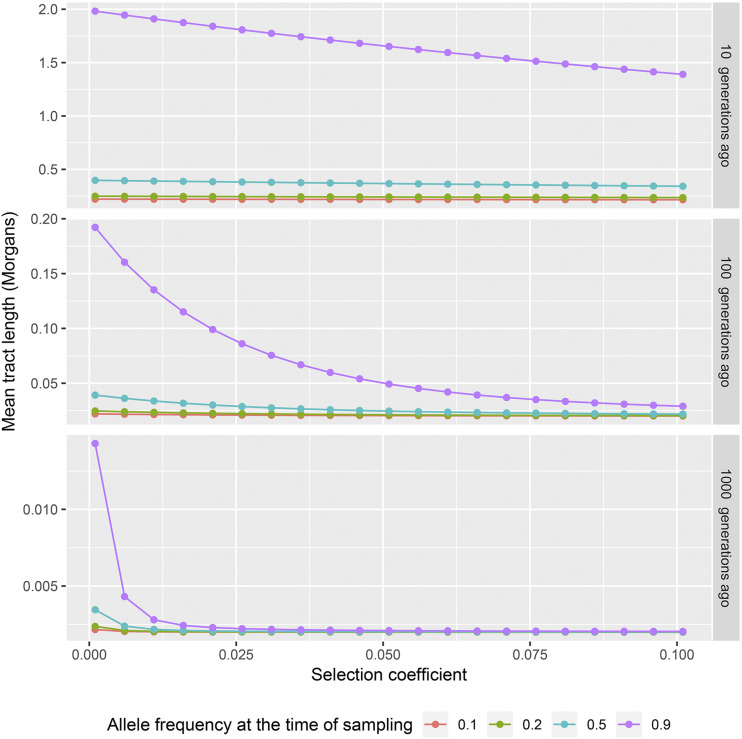
Dependence of the expected tract length on the strength of selection for different times of introgression. Different panels correspond to different times of introgression (10, 100 and 1000 generations respectively). Different colors correspond to different introgression proportion values (0.01, 0.05, 0.1).

The deterministic approach facilitates the calculation of expected tract lengths for a wide array of parameters. In Figure 2 we illustrate the dependency of the mean tract length on the admixture fraction given the time of introgression and selection coefficient. As expected, a larger admixture fraction results in longer expected tract lengths. In Figure 3 we illustrate the dependency on the selection coefficient given the time of introgression and admixture proportion. Again, stronger selection results in longer expected ancestry tract lengths.

The strength of selection has a stronger effect on the expected tract length than the admixture proportion. For example, consider an adaptive introgression model with time T=1000, selection coefficient s=0.01 and admixture proportion 0.025. The expected tract length is 0.0025 Morgans under this model. If we double the admixture proportion to 0.05, the expected tract length increases to 0.0029 Morgans, or approximately 13%. If on the other hand we increase the strength of selection by a factor of two, then the expected tract length increases by 41% and becomes 0.0036 Morgans.

**Figure 4:**
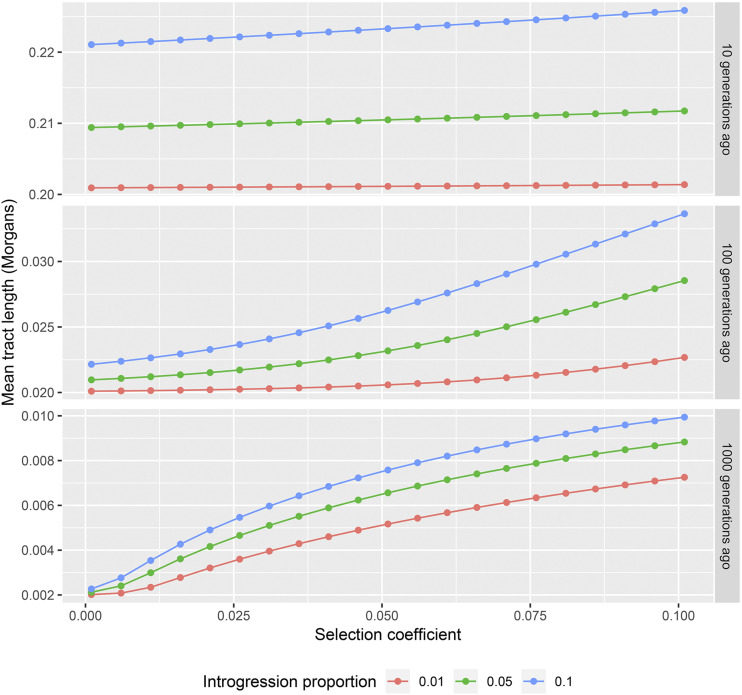
Dependence of expected tract length on the strength of selection conditioned on the allele frequency at the time of sampling. Different panels correspond to different times of introgression (10, 100 and 1000 generations respectively). Different colors correspond to different allele-frequency values at the time of sampling (0.1, 0.2, 0.5, 0.9).

The new approximation facilitates investigation of another important dependency, which would otherwise require prohibitively large numbers of simulations, namely the dependency of the expected tract length on the observed allele frequency. In Figure 4 we demonstrate that conditioning on the allele frequency at the time of sampling, stronger selection actually reduces the expected tract length. This is explained by the fact that stronger selection decreases (going backward in time) the allele frequency faster than weaker selection. Hence, the probability of recombining back to a haplotype that contains the selected allele following a recombination event is smaller for scenarios with stronger selection. More formally, assume that we compare two scenarios with selection coefficients $s_{1}$ and $s_{2}$ (all other parameters are the same), and that $s_{1}<s_{2}$. Let $\omega^{\left(i\right)}$ be the logistic trajectory for selection coefficient $s_{i}$. We condition on the observed allele frequencies at time $t_{0}$, hence $\omega^{\left(1\right)}\left(t_{0}\right)=\omega^{\left(2\right)}\left(t_{0}\right)$. Ancestral recombination occurs on a chromosome independent of the strength of selection. Assume that recombination occurred at time $t_{r}>t_{0}$. The expected allele frequency at time $t_{r}$, which is defined by the logistic function, is larger for the smaller selection coefficient: $\omega^{\left(1\right)}\left(t_{r}\right)>\omega^{\left(2\right)}\left(t_{r}\right)$. Hence, the chance that the remaining part of the chromosome coalesces back to a haplotype that contains the selected allele is higher for $s_{1}$.

### Denisovan introgression into Tibetans

A well-known example of adaptive introgression is Denisovan introgression into Tibetans which facilitated altitude adaptation by introducing an adaptive allele of EPAS1 affecting red blood cell production into the ancestors of modern Tibetans (Yi *et al.* 2010; Huerta-Sánchez *et al.* 2014). We wondered how selection might affect estimates of the time of introgression, conditioning on the present-day allele frequency of EPAS1 of 85% (Huerta-Sánchez *et al.* 2014) and an overal genomic admixture proportion of $\omega_{1}=0.06\pm 0.03\text{\%}$ (Sankararaman *et al.* 2016).

The admixture proportion is very small, and the logistic function will therefore be quite inaccurate. For a range of values of selection coefficient (s=0.005,0.006,…,0.02), we numerically estimated the expected trajectories (using the stochastic trajectory simulator) for the given admixture proportion. Given the frequency of the adapted allele in the modern population, the time since introgression is estimated as the time needed for the allele to reach this observed allele frequency following the expected trajectory.

We summarize the dependency of the introgression time on the strength of selection in Table 5 for the mean value $\omega_{1}=0.06\text{\%}$. It is clear from the table that dating the time of introgression from the tract length under the hypothesis of neutrality, would cause underestimation of the time of introgression by about 20−25%. For example, if the estimated tract length is 0.00117 Morgans, the corresponding introgression time without selection affecting the allele frequency is 1791 generations, or about 45000 years. However, taking selection into account, this tract length corresponds to selection coefficient s=0.006 and an introgression time of 2282 generations, or 57000 years ago. Therefore, analyses that seek to understand the timing of introgression events of adaptively introgressed alleles will be greatly facilitated by incorporating the dependency on the strength of selection on the expected tract length distribution. The best option for providing bounds for the time of introgression of single tracts, if these tracts cannot be assumed to be part of a larger introgression pulse that included other fragments, would be to jointly estimate the introgression time, the selection coefficient, and the time of introgression, using tract length, allele frequency, and other information such as local haplotype homozygosity and/or local genealogical information.

**Table 5:** **The effect of assumptions regarding the strength of selection on estimates of the time of the Denisovan introgression into Tibetans for EPAS1. We assume an initial introgression fraction of 0.06% (****Sankararaman *et al.* 2016****), and a present-day allele frequency for the EPAS1 allele of 85% in Tibetans (Huerta-Sánchez *et al.* 2014). Under deterministic approximation, the value of the selection coefficient then determines the time of introgression needed for the allele to reach the observed allele frequency at the time of sampling (present time). We calculate the expected length of introgressed Denisovan tracts overlapping EPAS1 allele for each such scenario. In the column “expected tract length (no selection)” we show the expected tract length for the given introgression proportion and time since introgression under hypothesis of no selection using formula 3. The last column shows the relative difference between the expected tract length estimated while taking into account selection and while ignoring it.**

Selection	Time(in generations)	Expected tract length	Expected tract length (no selection)	Relative error
0.005	2672	0.00101	0.00080	20.8%
0.006	2282	0.00117	0.00093	20.5%
0.007	2030	0.00130	0.00104	20.0%
0.008	1791	0.00146	0.00117	19.9%
0.009	1619	0.00160	0.00129	19.4%
0.01	1467	0.00175	0.00141	19.4%
0.011	1350	0.00190	0.00153	19.5%
0.012	1264	0.00201	0.00163	18.9%
0.013	1170	0.00217	0.00176	18.9%
0.014	1100	0.00230	0.00187	18.7%
0.015	1041	0.00242	0.00197	18.6%
0.016	983	0.00256	0.00209	18.4%
0.017	928	0.00270	0.00221	18.1%
0.018	883	0.00283	0.00232	18.0%
0.019	844	0.00296	0.00242	18.2%
0.02	806	0.00309	0.00253	18.1%

## Discussion

Adaptive introgression is an important and common phenomenon in evolutionary genetics (Hedrick 2013). In this paper we derived an accurate approximation model for the ancestry tract length distribution under adaptive introgression, near a selected site, with a single admixture pulse. This framework facilitated calculations of the distribution of tract lengths under a range of plausible adaptive introgression scenarios. However, we emphasize that future work should consider additional demographic models including continuous migration or multiple pulses. We demonstrated, perhaps counter-intuitively, that conditioned on the observed allele frequency, stronger selection produces shorter admixture tracts. Furthermore, as an example, we showed that selection should be taken into account when dating the well-known case of altitude adaptation in Tibetans through the introgression of EPAS1 gene from Denisovans. When ignoring the impacts of selection, the time since introgression might be underestimated by as much as 20–25% if conditioning on the allele frequency. If the allele frequency is allowed to be random, the effect would be opposite.

Our results illustrate that selection should be carefully considered and incorporated when studying adaptive introgression events. More generally, this framework opens new possibilities for understanding the timing of admixture and the strength of adaptive introgression across a wide range of populations. Our work therefore lays the groundwork for the development of new inference frameworks that can detect adaptive introgression using tract lengths and allele frequencies. Particularly, in light of the fact that adaptive introgression is common historically, and may be accelerated in the future by anthropogenic impacts such as climate change, *e.g.*, (Muhlfeld *et al.* 2014), developing a strong theoretical basis for understanding the impacts of adaptive introgression on patterns of genetic variation is crucial for interpreting the genomic consequences of admixture.
